# Mitochondrial DNA Polymorphism in HV1 and HV2 Regions and 12S rDNA in Perimenopausal Hypertensive Women

**DOI:** 10.3390/biomedicines11030823

**Published:** 2023-03-08

**Authors:** Wojciech Kwaśniewski, Aleksandra Stupak, Alicja Warowicka, Anna Goździcka-Józefiak, Jerzy Mosiewicz, Jolanta Mieczkowska

**Affiliations:** 1 Gynecology and Oncological Gynecology Department, Medical University of Lublin, 20-081 Lublin, Poland; 2 Obstetrics and Pregnancy Pathology Department, Medical University of Lublin, 20-081 Lublin, Poland; 3 Department of Molecular Virology, Adam Mickiewicz University in Poznan, 61-712 Poznan, Poland; 4 Internal Diseases Department, Medical University of Lublin, 20-081 Lublin, Poland

**Keywords:** mtDNA, nucleotide sequence, polymorphism, HV1 region, HV2 region, D-loop, menopause, arterial hypertension

## Abstract

Estrogens enhance cellular mitochondrial activity. The diminution of female hormones during menopause may have an effect on the mitochondrial genome and the expression of mitochondrial proteins. Hence, oxidative stress and the pro-inflammatory state contribute to the formation of systemic illnesses including arterial hypertension (AH). This study aimed to determine the types and frequency of mutations in the mitochondrial DNA (mtDNA) nucleotide sequence in the hypervariable regions 1 and 2 (HV1 and HV2) and the 12S RNA coding sequence of the D-loop in postmenopausal women with hypertension. In our study, 100 women were investigated, 53 of whom were postmenopausal and 47 of whom were premenopausal (53.9 ± 3.7 years vs. 47.7 ± 4.2 years, respectively). Of those studied, 35 premenopausal and 40 postmenopausal women were diagnosed with AH. A medical checkup with 24 h monitoring of blood pressure (RR) and heart rate was undertaken (HR). The polymorphism of the D-loop and 12S rDNA region of mtDNA was examined. Changes in the nucleotide sequence of mtDNA were observed in 23% of the group of 100 women. The changes were identified in 91.3% of HV1 and HV2 regions, 60.9% of HV1 segments, 47.5% of HV2 regions, and 43.5% of 12S rDNA regions. The frequency of nucleotide sequence alterations in mtDNA was substantially higher in postmenopausal women (34%) than in premenopausal women (10.6%), *p* = 0.016. A higher frequency of changes in HV1 + HV2 sections in postmenopausal women (30.2%) compared to the premenopausal group (10.6%) was detected, *p* = 0.011. Only postmenopausal women were found to have modifications to the HV2 segment and the 12S rDNA region. After menopause, polymorphism in the mtDNA region was substantially more frequent in women with arterial hypertension than before menopause (*p* = 0.030; 37.5% vs. 11.5%). Comparable findings were observed in the HV2 and HV1 regions of the AH group (35% vs. 11.5%), *p* = 0.015, in the HV1 segment (25% vs. 11.5%), *p* = 0.529, and in the HV2 segment, 12S rDNA (25% vs. 0%). More than 80% of all changes in nucleotide sequence were homoplasmic. The mtDNA polymorphisms of the nucleotide sequence in the HV1 and HV2 regions, the HV2 region alone, and the 12S RNA coding sequence were associated with estrogen deficiency and a more severe course of arterial hypertension, accompanied by symptoms of adrenergic stimulation.

## 1. Background

### 1.1. Mitochondrial DNA

The research of mitochondrial genome polymorphism began in the 1980s, when new tools in molecular biology made it possible to identify the mtDNA mitochondrial genome sequence [1]. The first mutation in the mitochondrial genome was described in 1988 and involved nucleotide 8344, a shift in the A/G sequence that causes myoclonic epilepsy accompanied with the presence of ragged red fibers in the muscles (MERRF syndrome, myoclonic epilepsy, and ragged red fibers) [2]. In human cells, the genetic material of the mitochondria constitutes 1% of the cell’s DNA [3]. MtDNA is a circular molecule containing 16,569 base pairs (bp) and is situated in the matrix of mitochondria. Each mitochondria carries four to ten copies of mtDNA [4,5]. The arrangement and structure of the mitochondrial genome parallels that of the bacterial genome. Moreover, mitochondrial ribosomes are prokaryotic in nature. Histone proteins are absent in mtDNA. Additional proteins related to mitochondrial DNA form structures known as nucleoids (nt). In both strands of mtDNA, 37 genes have been found in the mitochondrial genome (28 on the H (heavy) strand and 9 on the L (light) strand). Genes are tightly packed in mitochondrial DNA, and the sequences of some genes overlap and overlap (ATP8 and ATP6, ND4L, and ND4); there are few non-coding regions [6,7,8,9,10]. Mitochondria are also responsible for apoptosis’ so-called internal route. Along with this process is the release of cytochrome c and other proteins from the mitochondria. Released from the mitochondria, cytochrome c contributes to the synthesis of the Apoptosome protein complex [11]. Mitochondrial proteins belonging to the BCL-2 family, which are mostly localized in the outer mitochondrial membrane, and factor or endonuclease G are also implicated in the mitochondrial apoptotic process [12].

### 1.2. The D-Loop in mtDNA

The D-loop, which comprises 7% of the mitochondrial genome and regulates replication and transcription of mitochondrial genes, is an important non-coding region in mtDNA. It is composed of 1122 base pairs (from 16,024 nt to 567 nt mtDNA) and is located between the genes encoding the tRNA Pro and the tRNA Phe. The nucleotide sequences responsible for initiating gene transcription and mitochondrial genome replication have been found in this area [6,13,14,15]. In the D-loop region, there are also two hypervariable regions: the first (HV1) from 16,024 nt to 16,383 nt and the second (HV2) from 57 nt to 33 nt. In these places, the nucleotide sequence polymorphism is utilized in forensic investigations and medical diagnostics [16]. Additional variable regions in the D-loop of mtDNA, known as “hot” regions, are situated between 303 and 315 nucleotides and between 16,184 and 16,193 nucleotides [6,13,14,15]. In addition, the D-loop of mtDNA contained multiple point mutations, microsatellite instability alterations, and significant deletions. These modifications may influence mtDNA replication and the transcription of mitochondrial genes [17]. The mitochondrial genome encodes thirteen essential subunits of the oxidative phosphorylation (OXPHOS) machinery in the inner mitochondrial membrane [14]. There are 13 mitochondrial genes that encode respiratory chain-related proteins, 22 that encode transfer RNA (tRNA), and the remaining 2 encode ribosomal RNA (rRNA)—12SrRNA and 16SrRNA [13]. The respiratory chain of the mitochondria has 87 polypeptides. Both the mitochondrial genome (mtDNA) and the nuclear genome encode the proteins that construct the inner mitochondrial membrane respiratory chain (nDNA). The respiratory chain proteins encoded by mitochondrial DNA include complex I—NADH proteins. Complex II, which regulates succinate dehydrogenase, is encoded exclusively by nuclear DNA [18]. The non-coding region of the D-loop is responsible for the control of mtDNA replication and transcription. The straightforward arrangement of mtDNA renders this genome more susceptible to the action of mutagenesis agents, such as those that affect the nuclear genome. Thus, the same cell can contain both normal copies of mitochondrial DNA and their mutants. This occurrence is referred to as heteroplasmia [19].

### 1.3. Diseases Related to mtDNA

Several studies have demonstrated a link between genetic alterations in mitochondrial DNA and human disorders, including coronary heart disease, hypertension, diabetes, endometriosis, and cancer [20,21,22]. The hunt for the reasons of the aging processes has also drawn the attention of numerous researchers on the effect of oxidative stress on mtDNA alterations [23,24]. Mitochondria are the site of cellular energy transformations, and in particular the site of formation and storage of high-energy compounds. Tissues highly dependent on mitochondrial energy production are the heart, skeletal muscle, central nervous system, and kidney. Therefore, the cause of disturbances in the functioning of these organs may be a decrease in the efficiency of mitochondrial respiration caused by mtDNA mutations. The so-called mitochondrial illnesses are caused by mutations in the mitochondrial DNA [25]. Mutations in the structure and function of mitochondria disturb the proper functioning of these organelles and deregulate the apoptotic process, which is the root cause of numerous human disorders [26].

## 2. Material and Methods

### 2.1. Study Design

The purpose of this study was to determine the types and frequency of changes in the sequence of mitochondrial DNA nucleotides in the HV1 and HV2 regions and in the D-loop region encoding the 12S rDNA in postmenopausal women with essential arterial hypertension and hormonal abnormalities.

### 2.2. Study Population

The tests were conducted on 100 women, 53 postmenopausal and 47 premenopausal, at the Department of Internal Diseases, Outpatients Clinic, and Department of Gynecology at The 1st Independent Public Teaching Hospital of Medical University of Lublin, Poland.

### 2.3. Study Variables

Among 75% of the women in the study group, arterial hypertension was detected. In each case, the following tests were conducted: 1/medical examination consisting of an interview and physical examination; 2/measurement of blood pressure twice after rest; 3/24 h blood pressure and heart rate monitoring; and 4/investigation of mtDNA polymorphism in the HV1 and HV2 regions and the 12S rDNA coding region. During two medical appointments, a medical examination was conducted, and a questionnaire was used to collect data from the interview and medical examination. Blood pressure was measured twice in the examined women each time after a 10 min rest, using the mean value of these measurements.

Women with severe organic systemic diseases, previously diagnosed neoplastic diseases, previously diagnosed mitochondrial diseases, severe degenerative diseases, previously diagnosed secondary hypertension, previously diagnosed ischemic heart disease, a history of a heart attack or stroke, cardiomyopathies, congenital and acquired heart defects, previously diagnosed peripheral vascular diseases, diabetes, thyrotoxicosis, and thymoma were excluded from research.

### 2.4. Methods of Examination Conducted in Study

The postmenopausal phase was determined based on the patient’s medical history (menopausal symptoms such as hot flashes, increased sweating, and amenorrhea lasting over a year) and hormonal status (increase in FSH (follicle stimulating hormone) > 30 U/L in the blood serum).

Essential hypertension (AH) was diagnosed based on systolic blood pressure (RRs) 140 mmHg and/or diastolic blood pressure (RRr) 90 mmHg, as well as history (previously diagnosed and/or treated hypertension). Based on the systolic and diastolic blood pressure values, the European Society of Hypertension (ESC) distinguished arterial hypertension groups as optimal pressure, normal, high normal, arterial hypertension 1o, 2o, and 3o [27].

Every patient with arterial hypertension had outpatient 24 h monitoring of blood pressure and heart rate (ABPM). The hours beginning at 6 a.m. to 11 p.m. were taken as the waking and daytime hours, taking into account individual differences. Nighttime rest is 11 p.m. to 6 a.m. During the day, systolic and diastolic blood pressure were measured three times per hour, whereas at night, they were measured twice per hour. Systolic blood pressure (RRs) ≥ 135 mmHg during the day and diastolic blood pressure (RRr) ≥ 85 mmHg during the day were considered to be elevated, according to the recommendations of the European Society of Hypertension, and systolic blood pressure (RRs) ≥ 120 mmHg and diastolic pressure during the night were elevated (RRr) ≥ 70 mmHg.

The criteria for a sudden morning rise in blood pressure were increases in systolic and diastolic blood pressure of at least 10 mmHg. The increase is the difference between the mean nighttime measurements taken during sleep and the first two hours after awakening.

According to the magnitude of the decrease in systolic blood pressure, the following subgroups were distinguished among women with hypertension before and after menopause: 1. “dippers”—decrease in systolic blood pressure at night (RRs) compared to the day from 10 to 20%, 2. “non-dippers”—night systolic blood pressure (RRs) decrease in relation to the day to 10%, 3. “extreme dippers”—systolic blood pressure (RRs) drops by more than 20% between day and night, 4. “reverse dippers”—increase in systolic blood pressure (RRs) at night.

#### 2.4.1. Isolation of mtDNA Polymorphism in the Regions of HV1, HV2, and 12S rDNA

Blood was collected for genetic testing under standard fasting conditions. Blood cells (lymphocytes) of the patients were isolated using the QIAamp DNA Midi Kit (Qiagen, Hilden, Germany) according to the manufacturer’s isolation protocol [28]. The purity and concentration of DNA were analyzed spectrophotometrically (SynergyTM H1, BioTek, Santa Clara, CA, USA). The obtained DNA was suspended in EB buffer (10 mM TrisHCl pH 8.5) and stored at −20 degrees Celsius for future research.

#### 2.4.2. Analysis of the mtDNA D-Loop Mutation

MtDNA’s D-loop region was amplified with two PCR primer pairs. The primers had the following sequences: F4 5′ CACAGGTCTATCACCCTATTAACCA 3′ located at 4–28 bp, R599 5′ TTGAGGAGGTAAGCTACAT 3′ located at 599–581 bp, and F15974 5′ ACTCCACCATTAGCACCCAAA 3′ located at 15,974–15,994 bp; R16564 5′ TGATGTCTTATTTAAGGGGAACGT 3′ F4 and R16564 primers had previously been described [14]. In a 30 L reaction volume containing 1 PCR buffer, 1 M of each forward and reverse primer, 1.5 mM MgCl_2_, 200 M of each dNTP, and 1 U of Taq DNA polymerase, PCR amplifications were performed (Fermentas, Waltham, MA, USA). The PCR conditions were as follows: pre-denaturation at 95 °C for 15 min, followed by 40 cycles at 95 °C for 20 s, 57.6 °C for 45 s, 72 °C for 45 s, and a final extension at 72 °C for 6 min (for the F4 and R599 pair of primers); and pre-denaturation at 95 °C for 5 min, followed by 30 cycles at 95 °C for 30 s. The electrophoresis of PCR products amplified from D-loop mtDNA was carried out on 1.5% agarose gels. Following purification with the QIAquick PCR Purification Kit (Qiagen, Hilden, Germany) according to the manufacturer’s instructions, all PCR products were sequenced (in forward and reverse directions). The D-loop region’s nucleotide sequence was determined by comparing sequences to the Cambridge reference sequence (rCRS, NC 012920) [29].

#### 2.4.3. DNA Sequencing

The PCR-purified DNA samples were sequenced automatically. The Laboratory of Molecular Biology Techniques in Poznan, Poland, was tasked with completing this phase of research [28].

#### 2.4.4. Computer Evaluation

Chromas—Pro software (version 1.31) and DNAStar (MegAlign, Madison, WI, USA) were used to interpret the chromatograms of the sequenced DNA samples. The BLAST Align two success program and the BioEdit program were used to compare DNA sequences from different samples in order to detect mutations (NCBI database). NC 012920 was the sequence number selected from the databases as the reference sequence [29].

### 2.5. Ethics

Research procedures were in line with ethical standards for human experimentation. They were in accordance with the opinion of the Bioethics Committee of the Medical University of Lublin (No. KE-0254/185/2006, 26 October 2006) as well as the Helsinki Declaration of 1975 and its 2000 amendment. Each of the examined persons gave written informed consent to participate in the experiment.

### 2.6. Statistical Analysis

Using the Kolmogorov–Smirnov test (allowing for the assessment of the normality of the distribution), it was determined whether individual analyses including linear variables required parametric tests (Student’s *t*-test used in the comparisons of two independent groups if the assessed variables had a normal data distribution) or non-parametric tests (U-Mann–Whitney test used in the comparisons of 2 independent groups or the Spearman’s rank correlation test used to assess the correlation between 2 variables if the assessed variables had a distribution other than normal). In comparisons of linear variables, the mean was used as the measure of concentration, and the standard deviation as the measure of dispersion. As a result, the correlation coefficient R was calculated when evaluating the correlation. Moreover, in the case of nonlinear data (classified), their analysis was conducted using the logistic regression method, which included the calculation of Wald 2, odds ratios (ORs), and corresponding 95% confidence intervals (95%CI). In all analyses, alpha (*p*-value) values less than 0.05 were considered statistically significant.

## 3. Results

### 3.1. Age and Anthropometric Information Regarding the Respondents

A group of 100 non-smoking and non-alcohol-using women before (47 women) and after menopause (53 women), mean age 51.1 ± 5.0 years, mean body weight 70.3 ± 14.5 kg, BMI (body mass index) (kg/m^2^)- 27.2 ± 5.3, waist circumference (cm) 87.7 ± 13.7, and WHR waist/hip ratio 0.829 ± 0.061, was examined. The range of time since the last menstrual period was 0 to 7.9 years, with a mean of 4.5 to 7.9 years. No participant had ever utilized Hormone Replacement Therapy. The studied groups of pre- and postmenopausal women did not differ in terms of body weight, BMI, waist circumference, or WHR. A statistically significant difference existed between the age of the group of women studied before and after menopause. Women in the postmenopausal group were older than in the premenopausal group, and the difference was statistically significant (*p* < 0.01).

The average age of respondents with mtDNA polymorphism (before and after menopause) was 52.0 4.8 years and did not differ significantly from the average age of the other respondents (50.8 5.1 years; *p* = 0.131).

### 3.2. Analysis of the Sequences of the mtDNA HV1, HV2, and 12S RNA Regions

The nucleotide sequence of the most variable regions of the D-loop, HV1 and HV2, and the coding region of the 12S RNA of mtDNA were analyzed in total DNA isolated from the blood cells of 53 postmenopausal women and 47 premenopausal women who served as the reference group. A summary of the observed changes in the studied mtDNA regions in the entire group of women (in the pre- and postmenopausal period) is presented in Table 1.

In the group of 100 women (before and after the menopause), changes in the nucleotide sequence in the mtDNA segments studied were found in 23% of cases. The number of females and variations in the nucleotide sequence of mtDNA were as follows:

- With all mtDNA nucleotide sequence changes in the HV1 and HV2 regions and mtDNA 12S RNA coding sequence—23 women.
- With nucleotide sequence changes in the HV1 mtDNA region—14 women.
- With nucleotide sequence changes in the HV2 mtDNA region—11 women.
- With nucleotide sequence changes in the HV1 and HV2 regions of mtDNA—21 women.
- With changes in the coding sequence of nucleotides in the 12S RNA region of mtDNA—12 women.

The number of observed mtDNA changes in individual cases ranged from 1 to 18. They were present in 91.3% of the hypervariable regions (HV1 and HV2), and more frequently in the HV1 segment (60.9%) than the HV2 segment (47.5%). The number of observed changes in individual cases ranged from one to nine.

Changes were present in 43.5% of the 12S RNA coding region, where from two to five changes were observed. Changes in the mtDNA nucleotide sequence affected the HV1 segment in 60.9% of cases, the HV2 segment in 47.8% of cases, and the number of changes observed in individual cases ranged from one to nine. In 47.8% of cases with two to five lesions, mtDNA changes occurred in the 12S RNA coding sequence.

Changes in the Nucleotide Sequence of the HV1, HV2, and 12S RNA Regions of mtDNA are Presented in Table 2.

### 3.3. Homoplasmia/Heteroplasmia of mtDNA in the Study Group with Changes in the Mitochondrial Genome in the HV1 and HV2 mtDNA Regions

Most of the identified changes in the nucleotide sequence are homoplasmic (81.8% of respondents with changes in the HV1 region, 90.1% in the HV2 region, and 100% with changes in the region encoding the 12S RNA mtDNA). Heteroplasmic changes concerned the following nucleotides in the HV1 region: 16093TC, 16230AG, and 16286CT occurred in 18.2% of the subjects with changes in the HV1 region. In subjects with the 239TC nucleotide the changes in the HV2 region were detected in 9.9% cases.

### 3.4. Menopause

The frequency of nucleotide sequence changes in the mitochondrial genome of the studied postmenopausal women (34.0%) compared to the premenopausal period (10.6%) was significantly higher (*p* = 0.016). Both changes in the HV2 region and in the 12S RNA coding sequence occurred only in the studied postmenopausal women and were not observed in the studied group of premenopausal women. The frequency of nucleotide sequence changes in both HV1 and HV2 hypervariable segments in postmenopausal women was higher compared to the premenopausal period, and the difference was statistically significant (*p* = 0.011).

However, the incidence of mtDNA changes among the examined postmenopausal women in the HV1 segment only did not differ significantly in comparison to the group of premenopausal women. A comparison of the frequency of nucleotide sequence changes in different mtDNA segments in pre- and postmenopausal women is presented in Table 3.

Modifications in the nucleotide sequence in mtDNA occurred in women with hypertension (pre- and postmenopausal) in 25.3% of the subjects. Changes in the nucleotide sequence in mtDNA in postmenopausal women with arterial hypertension occurred in 37.5% of the respondents and in 11.5% of the premenopausal women. The difference was statistically significant (*p* = 0.030).

Table 4 presents a comparison of the frequency of mtDNA nucleotide sequence changes in the hypervariable sections of HV1 and HV2 and the coding sequence of 12S RNA with arterial hypertension in the premenopausal group and the postmenopausal group. The frequencies of nucleotide sequence changes in the HV1, HV2, and 12S rDNA mtDNA regions in pre- and postmenopausal hypertension are presented (37.5% vs. 11.5%, *p* = 0.03).

In subjects with premenopausal hypertension, no changes in mtDNA nucleotide sequence in the HV2 and 12S RNA segment were observed; hence, the influence of menopausal status on the incidence of these changes cannot be statistically expressed.

### 3.5. Parameters of Daily RR Monitoring in Patients with Arterial Hypertension Depending on Changes in mtDNA Nucleotide Sequence

**Figure 1:**
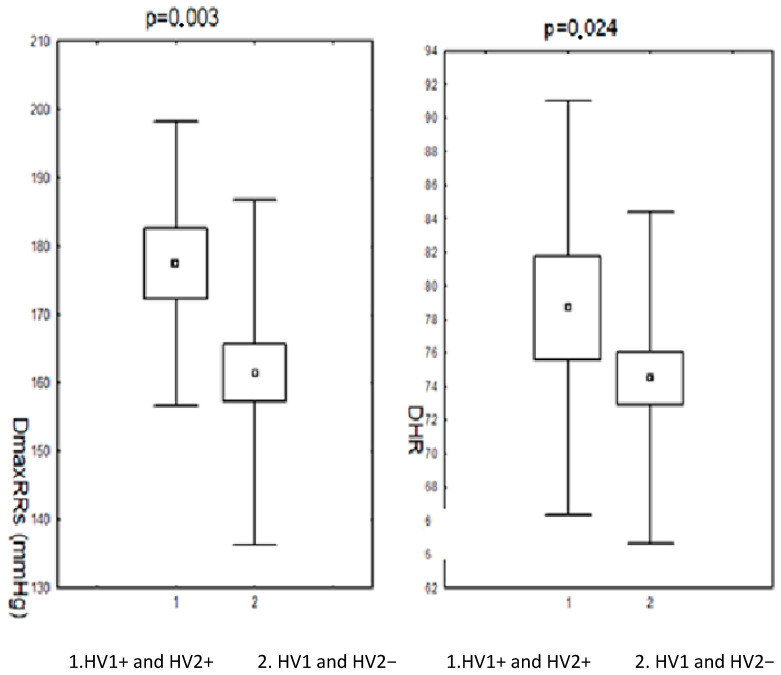
Results of 24 h measurements of systolic blood pressure during the day and heart rate in patients (pre- and postmenopausal) with arterial hypertension depending on changes in the nucleotide sequence in the HV1 and HV2 segment. DmaxRR—daytime maximum blood pressure; DHR—daily heart rate; HV1 and HV2+—positive for HV1 and HV2 changes; HV1 and HV2−—negative for HV1 and HV2 changes.

**Figure 2:**
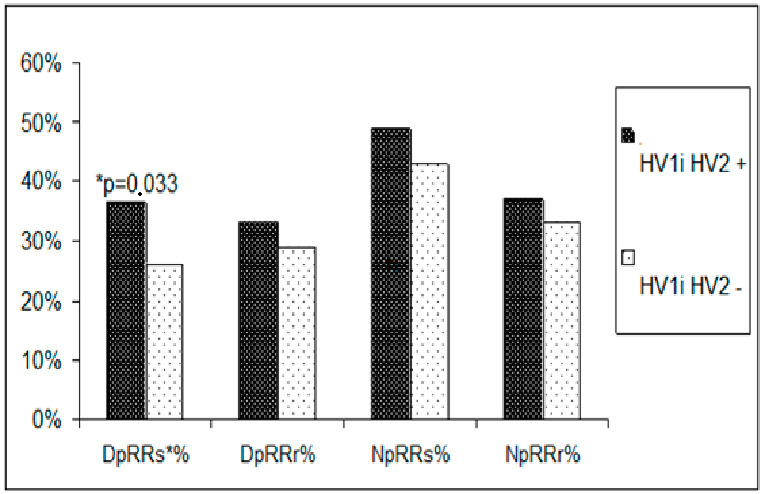
Comparison of the incidence of elevated blood pressure measurements obtained during 24 h blood pressure monitoring in pre- and postmenopausal women with hypertension. DpRRs%-percentage of increased systolic blood pressure values during the day; DpRRr%-percentage of increased diastolic blood pressure values during the day; NpRRs%-percentage of elevated systolic blood pressure values at night; NpRRr%-percentage of elevated diastolic blood pressure values at night. * denotes *p* value.

Changes in the HV1 and HV2 regions of mtDNA were accompanied by a modification of the parameters of the monitored RR. Statistically significantly higher maximum systolic RR and heart rate/min were observed, as well as a higher frequency of increased values of systolic RR during the day in the subjects (before and after menopause) in the group with nucleotide sequence changes in the HV1 and HV2 segments compared to the group without these changes. Parameters of daily RR monitoring in the subjects (before and after menopause) in the group with changes in the nucleotide sequence in the HV1 and HV2 regions and in the group without these changes are presented in Figure 1 and Figure 2.

### 3.6. Nucleotide Sequence Changes in HV1 and RR Monitoring

**Figure 3:**
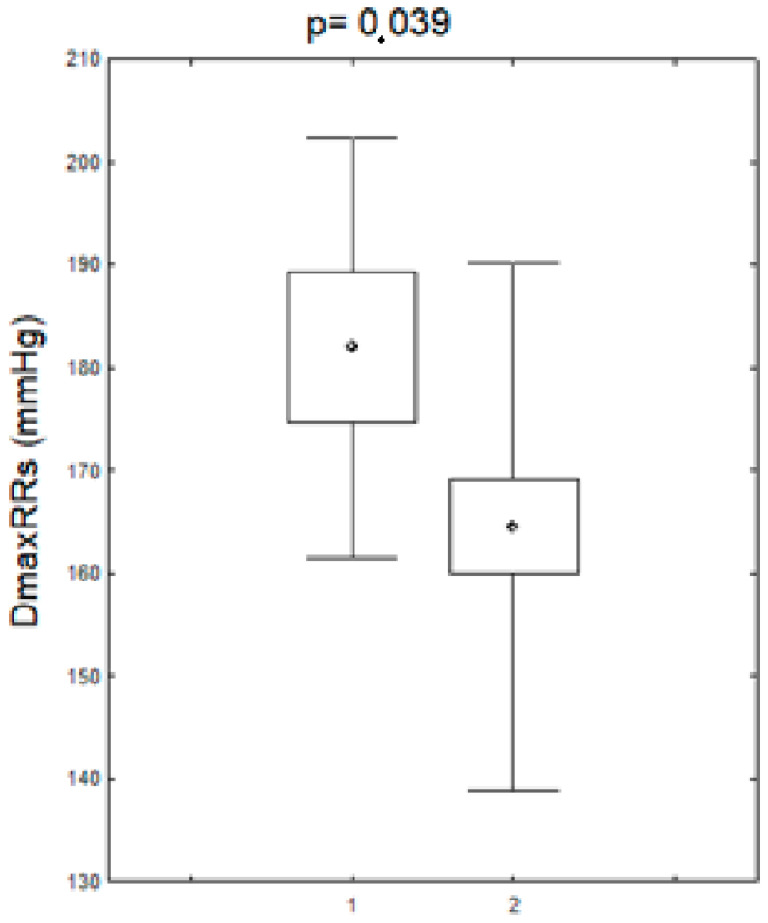
Maximum systolic blood pressure values during the day in patients (pre- and postmenopausal) with arterial hypertension with the changes in the HV1 regions. DDmaxRRs-daily maximum systolic blood pressure, HV1+,-positive for HV1 changes, HV1−,-negative for HV1 changes.

Alterations in the nucleotide sequence in the HV1 segment were associated with an increase in maximum daytime systolic blood pressure. The mean maximum systolic RR in the patients (before and after menopause) in the group with nucleotide sequence changes in the HV1 hypervariable region was statistically significantly higher compared to the group without these changes. The remaining parameters of the daily RR monitoring did not differ significantly in the studied women (before and after the menopause) between the group with changes in the nucleotide sequence in the HV1 segment as compared to the group without changes. A comparison of these parameters is presented in Figure 3.

### 3.7. Nucleotide Sequence Changes in HV2 and RR Monitoring

**Figure 4:**
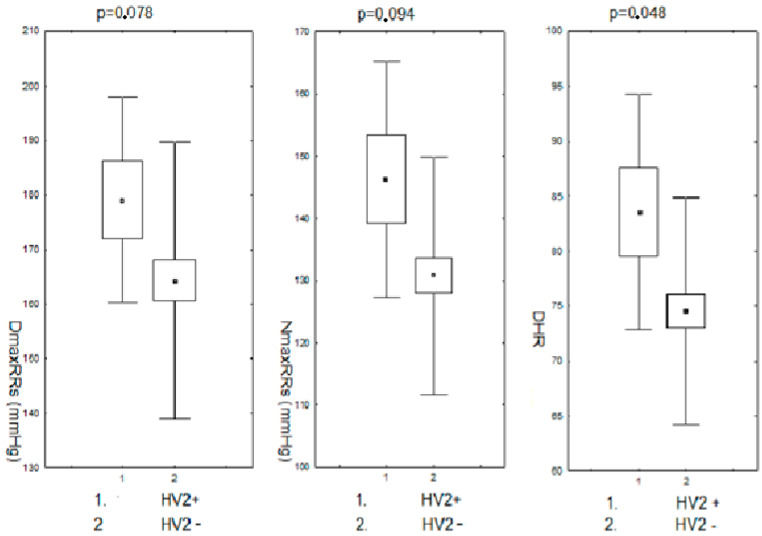
Maximum systolic blood pressure during the day and night and heart rate/min during the day in pre- and postmenopausal women with hypertension with changes in the HV2 regions. DDmaxRRs-daily maximum systolic blood pressure; NmaxRRs-nighttime maximum systolic blood pressure; DHR-daily heart rate; HV2+,-positive for HV2 changes; HV2−,-negative for HV2 changes.

Modifications in the nucleotide sequence in the HV2 mtDNA region were accompanied by slightly higher mean values of the maximum systolic RR both during the day and night, as well as significantly higher heart rate/min during the day. The comparison of RR monitoring parameters in the subjects (pre- and postmenopausal) in the group with changes in the nucleotide sequence in the HV2 hypervariable region to the group without changes is presented in Figure 4.

### 3.8. Nucleotide Coding Sequence Changes in 12S RNA and RR Monitoring

**Figure 5:**
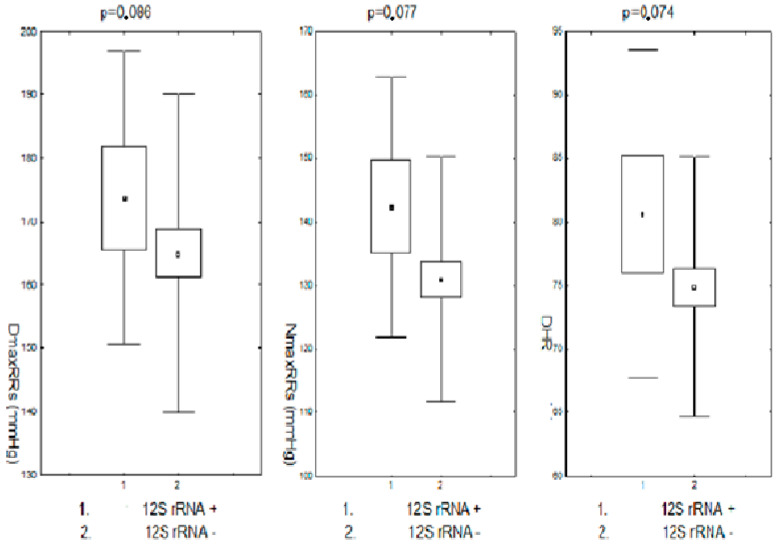
Maximum systolic blood pressure during the day and night and heart rate during the day in pre- and postmenopausal patients with arterial hypertension and in the region of the 12S RNA coding sequence. DDmaxRRs-daily maximum systolic blood pressure; NmaxRRs-nighttime maximum systolic blood pressure; DHR-daily heart rate; 12SrRNA+,-positive for 12SrRNA changes; 12SrRNA−,-negative for 12SrRNA changes.

Changes in the nucleotide sequence in the 12S RNA coding region were associated with the modification of the parameters of the monitored RR in their presence, but only in a way close to statistical significance. Maximum daytime and nighttime systolic RR and daytime heart rate were slightly higher in the group of subjects with nucleotide sequence changes in the coding region of the 12S RNA compared to the corresponding values in the group without these changes. The differences in each case were close to statistical significance. The other parameters of the monitored pressure showed no differences between these groups. These data are presented in Figure 5.

### 3.9. MtDNA Nucleotide Sequence Changes and Morning Rises in Blood Pressure

Nucleotide sequence changes in the HV1 and HV2 regions were associated with a slightly more frequent occurrence of morning increases in blood pressure, but without statistical significance. Both changes in the HV1 and HV2 regions separately and in the region of the 12S RNA coding sequence were not associated with more frequent morning increases in RR.

### 3.10. Nocturnal Drops in Blood Pressure and Changes in mtDNA Nucleotide Sequence

The percentage of reduction in systolic blood pressure at night in the premenopausal group with mtDNA nucleotide sequence changes was 15.7% and in the postmenopausal group with mtDNA changes was 11.1%, and the difference was not statistically significant, *p* = 0.180.

The frequency of the subgroups of hypertension—dippers, extreme dippers, reverse dippers, and non-dippers—was not dependent on changes in the nucleotide sequence in mtDNA. The difference in the incidence of these subgroups of hypertension in the subjects (pre- and postmenopausal) in the group with mtDNA nucleotide sequence changes compared to the group without these changes was statistically insignificant (*p* = 0.116). These data are presented in Table 5.

### 3.11. Summary

- Changes in the examined mtDNA segments occurred in 23% of women, more often after than before menopause, and in some areas they occurred only after menopause (12S rDNA, HV2). Most often they concerned the HV1 segment, but a slightly smaller percentage of patients showed changes in the HV2 segment and the 12S RNA coding sequence.
- Changes in mtDNA, regardless of localization, were associated with the course of arterial hypertension, greater and more frequent increases in systolic blood pressure, morning increases in blood pressure, and higher heart rate, suggesting adrenergic stimulation in these subjects.

## 4. Discussion

### 4.1. Rationale of the Study

The demographic structure of European societies has changed dramatically over the past decades. Data from 2020 show that 20.6% of people in European countries are over 65 [30]. The population aged 80 years or above in the EU’s population is projected to have a 2.5-fold increase between 2020 and 2100, from 5.9% to 14.6. This fact changes the tasks of medical care in European societies. Thus, women live nearly 10 years longer than men. The process of individual aging is easier to define in women due to the presence of menopause, which takes a woman from the period of full life and reproductive activity to the postmenopausal period leading to old age. Degenerative diseases, cardiovascular diseases, metabolic diseases, and neoplastic diseases occurring with the aging process determine the quality of life later in life. They have their genesis in molecular changes, in which mitochondrial dysfunctions play a non-negligible role, which may be related to the growth and accumulation of mutations within the mitochondrial genome [31].

### 4.2. mtDNA Polymorphism and Its Relation to Diseases and Aging

Single-nucleotide polymorphism studies allowed to determine genotypes responsible for a specific disease in monogenic diseases, and recently also genotypes with a high risk of multigene diseases [32,33]. One of the possible consequences of an mtDNA mutation—monogenic diseases—is rare. For example, various mtDNA mutations may be responsible for the monogenic mitochondrial disease—Leber’s hereditary optic neuropathy (LHON)—and 90% of patients have one of them: 11778G/A; 3460G/A; and 14484T/C. These mutations in the population are found with a frequency of 1/300 [34]. Changes in the nucleotide sequence in mtDNA are easy, easier than in the nuclear genome, which is associated with exposure of the mitochondrial genome to contact with continuously produced reactive oxygen species, as well as reduced mtDNA repair possibilities [35,36]. More frequent occurrence of mtDNA mutations in older age groups of patients was observed by Michikawa et al. [37]. Changes in the mtDNA nucleotide sequence occurred in the majority (in 57% of the studied patients) of the studied patients over 65 years of age, which, however, were not observed in the groups of younger patients. With age, the progressive increase in mtDNA mutations as a result of reaction to reactive oxygen species is secondary to lipid oxidation and the modification of mitochondrial proteins in the cell’s respiratory chain [38,39]. MtDNA mutations change the structure of the respiratory chain polypeptides encoded in the mitochondrial genome, reducing mitochondrial metabolic activity and the formation of high-energy compounds [40]. Respiratory chain enzymes encoded by altered mtDNAs disrupt electron transport, increase electron leakage from the respiratory chain, and increase the amount of free oxygen radicals produced, further damaging the mitochondria and creating a vicious circle effect. This mechanism leads to the deterioration of the functioning of organs and tissues during the aging process. Modification of apoptosis signaling secondary to mitochondrial damage has been observed in in vivo and in vitro studies. This thesis is confirmed by the results of studies by Wei et al. on skin fibroblasts [41]. The above-mentioned researchers observed greater disturbances in fibroblast bioenergetics in the elderly compared to younger people, which was assessed on the basis of a higher concentration of hydrogen peroxide, a high level of superoxide dismutase activity, and a decrease in the activity of cytochrome c oxidase, as well as the oxygen consumption rate in the older age group. At the same time, a decrease in pyruvate dehydrogenase (PDH) expression and an increase in lactate dehydrogenase kinase were observed.

### 4.3. Study Subjects and Comparison of Results with Previous Studies

Despite the relationships of mtDNA polymorphism with age repeatedly described in the scientific literature, our studies did not show significant differences in age between the groups of women studied with changes in nucleotide sequences in the D-loop and without mtDNA changes. MtDNA changes with age, hence, we studied women over 40 (between 40–60 years old). The restricted age range of the research group may have prevented age disparities between women with mtDNA nucleotide sequence alterations and those without. Disturbances in the physiological functions of mitochondria may depend not only on the direct effect of the mutation on the respiratory chain, but may also occur secondary to the existing multigene disease and dysfunction of the mitochondrial respiratory chain in its course. However, it should be emphasized that certain mtDNA mutations may be beneficial. There are publications regarding changes in nucleotide sequences in mtDNA accompanying longevity. Studies by Kokaze et al. found that the mtDNA 5178 C/A polymorphism, which is associated with longevity, may prevent the onset of diabetes [42]. It has been shown that this genotype reduces the number of mtDNA mutations in oocytes, as well as the rate of mtDNA mutation formation and their accumulation in somatic cells in Japanese centenarians. The mtDNA 5178 C/A polymorphism not only prevents diabetes but is probably responsible for inhibiting the development of myocardial infarction [43]. Zhang et al., in the Italian population, studied the frequency of the C150T mutation located near the sequences responsible for mtDNA heavy strand synthesis. Its occurrence was more frequent in older age groups [44]. It appeared in approximately 17% of people (33/52) aged 99–106, while in younger people (aged 18–98) only in 3.4% (3/117).

Howell et al. and others found that mutations associated with multigenetic illnesses commonly occur in the D-loop region, a 1122 bp non-coding stretch of mtDNA containing two hypervariable regions, HV1 and HV2 [45,46]. This area has higher mtDNA polymorphism than others. Del Bo et al. found more mutations in the HV1 and HV2 hypervariable regions of the D-loop than other mtDNA segments in aged people [47]. Mutations in D-loop mtDNA nucleotide sequences, which occur often, disrupt mitochondrial genome replication and transcription. A single-nucleotide polymorphism in the D-loop and 12S RNA coding sequence of mtDNA was detected in 23% of our respondents. Like the aforementioned authors, we found more frequent changes in the hypervariable segments HV1 and HV2 of the D-loop, which occurred in 21.0% of respondents, somewhat more often in the HV1 segment (14.0% of respondents) than in HV2. In the HV1 area, 85.7% of mutations were homoplasmic and non-coding, and just one patient implicated nucleotide 16319, the beginning site for mtDNA synthesis and light strand engaged in replication. Nucleotide sequence alterations in the HV2 region were homoplasmic in 90.1% of instances, connected to the transcription factor binding site in CBS3 (conserved block) in 36%, and occurred in nearly 30% of responses. The 12S rDNA region has 10% non-coding nucleotides, 90% 1438AG and 750AG, and 20% 930GA. Rydzanicz et al. found polymorphisms in the 12S rDNA region (G709A, G750A, G930A, T1243C, T1420C, and G1438A) at a frequency greater than 1% [48]. This study found two mtDNA polymorphisms in the HV2 hypervariable section of the mitochondrial genome that have not been previously reported. A postmenopausal woman with arterial hypertension and metabolic syndrome had a polymorphism. The non-coding nucleotide 340C/A in the H strand origin region between the DNA replication primer and the CBS3 block was changed. The second nucleotide sequence change included the non-coding nucleotide 362T/C and the conserved CBS3 block. Our postmenopausal control patient showed another polymorphism. It was in the mtDNA 12S RNA coding sequence and associated with the non-coding nucleotide 812A/C. Most of the other alterations in the investigated population include mutations associated with multigene illnesses, coronary artery disease, hypertension, diabetes, and neoplastic diseases, according to the literature [26,49,50]. In multigene diseases, the disease process in such cases is not caused by a single change in nucleotide sequences in mtDNA, but by changes in many genes. The presence of mtDNA polymorphisms is not a prerequisite for clinical symptoms of the disease, and changes in the mitochondrial genome occur only in some patients with clinical symptoms of the disease. Homoplastic mutations, which do not always translate into the phenotype of clinical disease symptoms, are often diagnosed at random. This fact is explained by many authors by the direct influence on the mitochondrial genome of the nuclear genome and the influence of epigenetic factors, while the disclosure of heterozygous mutations is conditioned by the proportion of mutated and normal mtDNA [51,52]. Most polymorphisms occur as a variant of the genotype not associated with the occurrence of a given disease, and changes in nucleotide sequences are often located only in the vicinity of genes responsible for a given disease entity. As a result of changes in mtDNA, the same mutation may cause various sets of clinical symptoms or be asymptomatic. In summary, mitochondrial mutations may cause phenotypic effects that are difficult to predict and may occur as pathogenic or only potentially pathogenic mutations.

The study found that alterations in mtDNA in HV1, HV2, and 12S rDNA may impact arterial hypertension by increasing blood pressure day and night and heart rate, suggesting an increased adrenergic system tone in these people. The HV2 segment (239TC, 243AG, 247GA, 250TC, 260GA, 277CT, and 284CT) alterations mostly affected transcription factor binding sites and non-coding nucleotides in the 12S RNA and HV1 coding regions. According to Pejovic et al., the nucleotide sequences in the hypervariable D-loop regions that replicate mtDNA and the degree of transcription factor binding can alter mtDNA synthesis and cell number [53]. The examined women’s HV2 region alterations, which impact mtDNA synthesis and transcription factor binding, may affect blood pressure. Several studies link hypertension to mitochondrial metabolism and free oxygen radicals [49,54]. On the other hand, the authors of experimental studies describe various forms of damage to the mitochondrial respiratory chain, leading to an increase in the production of reactive oxygen species that affect the course of hypertension. The source of reactive oxygen species in blood vessels are vascular endothelial cells, fibroblasts, and vascular smooth muscle, in which NAD (P) H or NADH oxidase (nicotinamide adenine dinucleotide in reduced form) catalyzing the reduction of oxygen causes the formation of O2 - and large amounts of other free radical oxygen. NAD (P) H oxidase activation occurs under the influence of TNF-α, angiotensin, and nitric oxide synthase. Hydrogen peroxide is a vasoactive compound with vasoconstrictor properties. According to Rubanyi et al., peroxygen hydrogen chloride in reaction with nitric oxide can transform into peroxynitrite anion (ONOO–), which reduces the availability of nitric oxide and thus contributes to the development of hypertension [55]. According to Pryor et al., hydrogen peroxide directly affects the opening of potassium and calcium channels, and therefore is also responsible for vasodilation. In turn, nitric oxide synthase is a source of not only NO, but also O_2_, which reduces the availability of NO [56]. According to the authors cited above, the balance between NO and O_2_, is essential for the damage to the vessel wall, the state of vascular tone, and the development of arterial hypertension. The production of large amounts of free oxygen radicals activates the tyrosine phosphatase and tyrosine kinase pathways, influences the expression of transcription factors and mitogen-activated protein kinases, and changes the activity of ion channels. ROS directly increases the concentration of calcium ions in the cell, leading to vessel wall dysfunction and remodeling. The changes in mtDNA observed in the group of women we have studied, accompanying higher blood pressure values, are probably the result of damage to the mitochondria and the formation of ROS. Many of the available publications on mitochondrial mutations in studied patients with maternal hypertension refer to Asian populations [57,58,59,60,61]. Various degrees of arterial hypertension recognized in the presence of mutations were observed: mutation 4435A > G, with a 30% reduction in mitochondrial metabolism and mitochondrial tRNA transcription (Met); mutation 4263A > G, located at the site of transcription for isoleucine (5’ end of tRNA (Ile)), which decreased the efficiency of the tRNA replication process by about 46%; the 4401A > G nucleotide mutation located directly at the 5’ end of the tRNA (Met) and tRNA (Gln), with a reduction in the mitochondrial translation index and a reduction in the mitochondrial respiratory efficiency index; and mutation T3308C on the dehydrogenase subunit (ND1), in which the translation initiating amino acid-methionine has been replaced with tyronine in ND1, with the alteration of RNA precursor strand processing or destabilization of ND1-mtRNA dehydrogenase.

The results of this study and other investigations suggest that alterations in the mtDNA nucleotide sequence may cause arterial hypertension. A shortage of postmenopausal sex hormones may damage the mitochondrial respiratory chain, causing mtDNA alterations and hypertension as a multigene illness. The literature shows multigene alterations in mtDNA coding and non-coding nucleotides in arterial hypertension, comparable to our findings [62]. The function of mtDNA D-loop hypervariable regions in arterial hypertension was examined by Liu et al. Changes in the HV2 area 152T-> C, 182C-> T, and 247G-> A and HV1 segment 16187C-> T, 16189T-> C, 16264C-> T, 16270C-> T, and 16311T-> C predispose to essential hypertension. According to these authors, the development of hypertension does not correlate to the severity of the mutation’s influence on the illness’s clinical picture, and the environment and nuclear gene modifiers in these individuals also modify the mutation’s effect on the disease. In a Japanese population investigation on the association between mtDNA alterations and arterial hypertension, Soji et al. found that the mtDNA genotype 16223TC is more prevalent in hypertensive patients than in those without hypertension and is associated with higher hypertension risk [63]. They found no connection with other genotypes, including C16362T. As in this study, studies on the mitochondrial genome generally focused on single or many gene coding or non-coding agents and their link with disease entities, such as hypertension.

### 4.4. Strength and Limitations of the Study

One of the limitations of our research is that it involved women from 40 to 60 years of age, who could be expected to have changes in mtDNA with age. Another issue perhaps would be the small age range of the study group, which meant that the differences in the age of the studied women with changes in mtDNA nucleotide sequences compared to the patients without changes did not occur.

The investigators did not investigate other factors leading to hypertension, such as nutrition, which is a drawback of the study. Obesity can induce hypertension and therefore have an impact on changes in the mitochondrial genome sequence. 

Our discovery may have clinical relevance in that Hormone Replacement Therapy may have a protective effect on mtDNA mutation. Several experimental medicines have already reached the clinical phase with extremely promising findings, yet the likelihood of enrolling patients in clinical trials is limited.

## 5. Conclusions

Changes in the HV1 and HV2 segments of mitochondrial DNA are accompanied by a more severe course of arterial hypertension with symptoms of adrenergic stimulation (higher maximum systolic pressure during the day and night, more frequent increases in systolic pressure, more frequent morning increases in blood pressure, and higher average heart rate).

The course of arterial hypertension in the mtDNA polymorphism group was more severe than in the patients without these changes, and at the same time there were symptoms of increased tension of the adrenergic system (slightly higher maximum systolic RR during the day and night and higher heart rate during the day).

In the HV2 hypervariable segment the mtDNA changes not yet described in the literature were detected: in the non-coding nucleotide 340 C/A (in the heavy strand H region) and in the non-coding nucleotide 362 T/C (in the region including the conservative block CBS3) in a postmenopausal woman with hypertension and metabolic syndrome and in the non-coding nucleotide 812 (AC) (region 12S RNA) in a postmenopausal control patient.

## Figures and Tables

**Table 1:** List of changes in mtDNA in the HV1, HV2, and 12S RNA regions in the entire study group of women and in the group of pre- and postmenopausal women.

mtDNA Region	Nucleotide Position in mtDNA	Sequence according to the MITOWEB Database *	Change	% Changes in mtDNA in the Entire Study Group	% Changes in mtDNA in the Premenopausal Study Group	% Changes in mtDNA in the Postmenopausal Study Group
MT-HV2	73 (-)	A	A-G	5 (5%)	0	5 (9.4%)
MT-OHR	127 (-)	T	T-C	1 (1%)	1 (2.1%)	0
MT-HV2	146 (-)	T	T-C	1 (1%)	0	1(1.9%)
MT-HV2	150 (-)	C	C-T	2 (2%)	0	2 (3.8%)
MT-OHR	152 (-)	T	T-C	2 (2%)	0	2 (3.8%)
MT- HV2	195 (-)	T	T-C	3 (3%)	0	3 (5.7%)
MT-OHR	199 (-)	T	T-C	1 (1%)	0	1(1.9%)
MT-OHR	204 (-)	T	T-C	1 (1%)	0	1(1.9%)
MT-OHR	207 (-)	G	G-A	1 (1%)	0	1 (1.9%)
MT-TFX	239	T	T-C	1 (1%)	0	1 (1.9%)
MT-TFX	243	A	A-G	1 (1%)	0	1 (1.9%)
MT-TFX	247	G	G-A	1 (1%)	0	1 (1.9%)
MT-OHR	250	T	T-C	1 (1%)	0	1 (1.9%)
MT-TFX	260	G	G-A	1 (1%)	0	1 (1.9%)
MT-OHR	263 (-)	A	A-G	9 (9%)	0	9 (17.0%)
MT-OHR	264 (-)	C	C-T	1 (1%)	0	1 (1.9%)
MT-OHR	266 (-)	T	T-G	1 (1%)	0	1 (1.9%)
MT-TFY	277	C	C-T	1 (1%)	1	0
MT-TFY	284	C	C-T	1 (1%)	1 (2.1%)	0
MT-TFY	295	C	C-T	1 (1%)	0	1 (1.9%)
MT-CBS2	310	C	C-T	3 (3%)	0	3 (5.7%)
MT-CBS2	311	C	C-T	2 (2%)	0	2 (3.8%)
MT-HPR	319	T	T-C	1 (1%)	0	1 (1.9%)
MT-OHR	338 (-)	C	C-T	1 (1%)	0	1 (1.9%)
MT-OHR	340 (-)	C	C-A	1 (1%)	0	1 (1.9%)
MT-CBS3	362	T	T-C	1 (1%)	0	1 (1.9%)
MT-HV3	480	T	T-C	1 (1%)	0	1 (1.9%)
MT-3H	385	A	A-G	1 (1%)	0	1 (1.9%)
MT-HV3	477 (-)	T	T-C	1 (1%)	0	1 (1.9%)
MT-HV3	489 (-)	T	T-C	1 (1%)	0	1 (1.9%)
MT-RNR1	709 (-)	G	G-A	1 (1%)	0	1 (1.9%)
MT-RNR1	750 (-)	A	A-G	10 (10%)	0	10 (18.9%)
MT-RNR1	812 (-)	A	A-G	1 (1%)	0	1 (1.9%)
MT-RNR1	930 (-)	G	G-A	2 (2%)	0	2 (3.8%)
MT-RNR1	961 (-)	T	T-G	1 (1%)	0	1 (1.9%)
MT-RNR1	1189	G	G-C	1 (1%)	0	1 (1.9%)
MT-RNR1	1438 (-)	A	A-G	10 (10%)	0	10 (18.9%)
MT-HV1	16,069 (-)	C	C-T	2 (2%)	0	2 (3.8%)
MT-7SDNA	16,093 (-)	T	T-C	1 (1%)	1 (2.1%)	0
MT-HV1	16,126 (-)	T	T-C	5 (5%)	1	4
MT-HV1	16,129 (-)	G	G-A	1 (1%)	0	1 (1.9%)
MT-HV1	16,140 (-)	T	T-C	1 (1%)	0	1 (1.9%)
MT-7SDNA	16,145 (-)	G	G-A	2 (2%)	1 (2.1%)	1 (1.9%)
MT-HV1	16,147 (-)	C	C-T	1 (1%)	1 (2.1%)	0
MT-HV1	16,174	C	C-T	1 (1%)		1 (1.9%)
MT-DLOOP	16,176 (-)	C	C-G	1 (1%)	1 (2.1%)	0
MT-HV1	16,189 (-)	T	T-C	1 (1%)	0	1 (1.9%)
MT-DLOOP	16,192 (-)	C	C-T	1 (1%)	0	1 (1.9%)
MT-HV1	16,222 (-)	C	C-T	1 (1%)	0	1 (1.9%)
MT-HV1	16,223 (-)	C	C-T	3 (3%)	1 (2.1%)	2
MT-DLOOP	16,224 (-)	T	T-C	1 (1%)	0	1 (1.9%)
MT-DLOOP	16,230 (-)	A	A-G	1 (1%)	1 (2.1%)	0
MT-DLOOP	16,256 (-)	C	C-T	1 (1%)	0	1 (1.9%)
MT-HV1	16,261 (-)	C	C-T	2 (2%)	0	2 (3.8%)
MT-DLOOP	16,263 (-)	T	T-C	1 (1%)	1 (2.1%)	0
MT-HV1	16,264 (-)	C	C-T	1 (1%)	0	1(1.9%)
MT-HV1	16,266 (-)	C	C-A	1 (1%)	0	1 (1.9%)
MT-DLOOP	16,270 (-)	C	C-T	3 (3%)	0	3 (5.7%)
MT-DLOOP	16,286 (-)	C	C-T	1 (1%)	1 (2.1%)	0
MT-HV1	16,288 (-)	T	T-C	1 (1%)	0	1 (1.9%)
MT-HV1	16,292 (-)	C	C-T	1 (1%)	0	1 (1.9%)
MT-HV1	16,294 (-)	C	C-T	2 (2%)	1 (2.1%)	1 (1.9%)
MT-HV1	16,296 (-)	C	C-T	2 (2%)	1 (2.1%)	1 (1.9%)
MT-DLOOP	16,298 (-)	T	T-C	1 (1%)	1 (2.1%)	0
MT-HV1	16,304 (-)	T	T-C	2 (2%)	1 (2.1%)	1 (1.9%)
MT-HV1	16,311 (-)	T	T-C	3 (3%)	0	3
MT- HPR	16,319	G	G-A	1 (1%)	0	1 (1.9%)
MT-HV1	16,362 (-)	T	T-C	2 (2%)	0	2
MT-DLOOP	16,390 (-)	G	G-A	1 (1%)	1 (2.1%)	0
MT-DLOOP	16,391 (-)	G	G-A	1 (1%)	0	1 (1.9%)
MT-DLOOP	16,497 (-)	A	A-G	1 (1%)	0	1 (1.9%)
MT-DLOOP	16,519 (-)	T	T-C	8 (8%)	4 (8.5%)	4 (7.6%)
MT-DLOOP	16,526 (-)	G	G-A	1 (1%)	0	1 (1.9%)

* According to the MITOWEB database, www.mitomap.org (accessed 31 January 2023) [28]: (-), non-coding nucleotide MT-RNR1 ribosomal RNA (12S RNA mtDNA); MT-HV1, hypervariable segment 1 (from nucleotide 16,024 to 16,365); MT-HV2, hypervariable segment 2 (from nucleotide 73 to 340); MT-HV3, hypervariable segment 3; MT-OHR, H-strand origin; MT-CBS2, conserved sequence block 2 (conserved sequence box); MT-CBS3, conserved sequence block 3 (conserved sequence box); MT-TFX, mtTF1 binding site; MT-TFY, mtTF1 binding site; MT-HPR, replication primer; MT-7SDNA-7SDNA; MT-3H, mt3 H-strand control element.

**Table 2:** Distribution of nucleotide changes in HV1, HV2, and 12S RNA regions of mtDNA of all patients.

Location	Others	Modifications	Cases (%)
HV1		16519TC	64.2
		16126TC	35.7
		16311TC, 16223CT, and 16270CT	21.4
		16069CT, 16145GA, 16261CT, 16294CT, 16296CT, 16304TC, and 16362TC	14.3
		16093TC, 16129GA, 16140TC, 16147CT, 16174CT, 16176CG, 16189TC, 16192CT, 16222CT, 16224TC, 16230AG, 16256CT, 16263TC, 16264CT, 16266CA, 16286CT, 16288TC 16292CGA, 16319GA, 16291GA, 16292TCGA, 16319GA, 16319GA, and 16319GA	7.1
HV2		263AG	81.8
		73AG	45.5
		195TC	27.3
		150CT, 152TC	18.2
	Transcription factor binding site	239TC, 243AG, 247GA, 250TC, 260GA, 277CT, 284CT, and 295CT	36.4
	Coding region—CBS3	310CT	27.3
	Coding region—CBS3	311CT	18.2
12S RNA		1438AG, 750AG	90
		930GA	20
		709GA, 812AG, 961TG, and 1189GC	10
	Section MT3H	385AG	1
	Section HV3	477TC	1
	Section HV3	489TC	1
	Section HV3	480TC	1

**Table 3:** Comparison of the frequency of nucleotide sequence changes in different mtDNA segments in the studied groups of pre- and postmenopausal women.

Changes	Premenopausal Women	Postmenopausal Women	Χ^2^ Walda	Odds Ratio	Range	*p*-Value
	47 (47%)	53 (53%)				
mtDNA	5 (10.6%)	18 (34.0%)	6.0	3.9	1.29–11.8	0.016
HV1	5 (10.6%)	9 (17.0%)	0.55	1.56	0.48–5.1	0.457
HV2	0 (0%)	11 (20.8%)	n/a			
HV1+HV2	5 (10.6%)	16 (30.2%)	6.4	4.6	1.4–15.1	0.011
12S DRNA	0 (0%)	12 (22.6%)	n/a			

**Table 4:** The frequency of nucleotide sequence changes in various mtDNA regions in the group of women with arterial hypertension depending on the menopausal status.

	Premenopausal Group	Postmenopausal Group	Χ^2^ Walda	Odds Ratio	Range	*p*-Value
Number of cases	35 (74.5%)	40 (75.5%)				
Changes HV1, HV2, 12S rDNA	4 (11.5%)	15 (37.5%)	4.7	3.9	1.1–13.8	0.030
HV1	4 (11.5%)	10 (25%)	0.39	1.5	0.39–5.9	0.529
HV2	0 (0%)	10 (25%)	n/a			
HV1 + HV2	4 (11.5%)	14 (35%)	5.9	5.4	1.4–21.6	0.015
12S rDNA	0 (0%)	10 (25%)	n/a			

**Table 5:** The incidence of hypertension subgroups (dippers, extreme dippers, reverse dippers, and non-dippers) depending on the presence of nucleotide sequence changes in mtDNA.

	All Changes +	All Changes −	*p*-Value
AH (number)	18	57	
“dippers”	52.9%	34.2%	0.166
“non-dippers”	29.4%	53.7%	
“extreme dippers”	5.8%	7.3%	
“reverse dippers”	11.8%	4.9%	

## Data Availability

The datasets used and/or analyzed during the current study are available from the corresponding author on reasonable request. All of the mentioned genetic polymorphisms in our manuscript are already deposited in dbSNP at https://www.ncbi.nlm.nih.gov/snp/ (accessed on 31 January 2023). The reference sequence selected from the databases was the sequence number NC_012920.

## Abbreviations

AH	arterial hypertension
Bp	base pair
HV1	hypervariable region 1
HV2	hypervariable region 2
mtDNA	mitochondrial genome
nDNA	nucleotide DNA
Nt	nucleotide
RR	blood pressure
